# Effect of sodium hypochlorite gel on bacteria associated with periodontal disease

**DOI:** 10.1007/s00784-023-05446-9

**Published:** 2024-03-02

**Authors:** Delia Irani, Gert Jungbauer, Anton Sculean, Sigrun Eick

**Affiliations:** School of Dentistry, Department of Periodontology, University of Bern, Freiburgstrasse 7, CH-3010 Bern, Switzerland

**Keywords:** Periodontal biofilm, Anti-biofilm agent, Sodium hypochlorite, Hyaluronic acid

## Abstract

**Objectives:**

An adjunct in non-surgical periodontal therapy might be sodium hypochlorite (NaOCl)–based agents. The purpose of the present in vitro study was to get deeper knowledge on the influence of different parameters as time after mixing, pH, and chemical composition of an amino acid 0.475% NaOCl (AA-NaOCl) gel consisting of two components on its anti-biofilm activity.

**Materials and methods:**

Six-species biofilms were cultured for 5 days, before AA-NaOCl gel was applied. In the different series, the influence of the time after mixing of the two components before application, of the concentration of NaOCl in the gel mixture, of the pH of the gel mixture, and of an exchange of the amino acid component by hyaluronic acid (HA), was analyzed.

**Results:**

Mixing time point experiments showed that the AA-NaOCl gel is capable of statistically significantly reducing colony-forming unit (cfu) counts up to 30 min after mixing, but only up to 20 min after mixing the reduction was more than 2 log10 cfu. The pH experiments indicate that a reduced pH results in a reduced activity of the NaOCl formulation. NaOCl concentrations in the formulation in the range from 0.475 to 0.2% provide adequate activity on biofilms. A HA/NaOCl gel was equally active against the biofilm as the AA-NaOCl gel.

**Conclusion:**

Mixing of the components should be made in a timeframe of 20 min before applications. An optimization of the composition of the NaOCl formulation might be possible and should be a topic in further in vitro studies.

**Clinical relevance:**

The AA-NaOCl gel formulation can be mixed up to 20 min before application. Further, the study indicates that the composition of the NaOCl gel formulation can be optimized.

## Introduction

Periodontitis is a bacterially induced chronic inflammatory disease, where an imbalance of the innate immune defense system markedly contributes to the destruction of the tooth-supporting tissue [1]. Microorganisms are organized in biofilms; the subgingival biofilms consist of hundreds of species [2, 3]. Among the bacteria more present in periodontitis than in periodontal health are *Treponema denticola*, *Porphyromonas gingivalis*, *Tannerella forsythia*, *Filifactor alocis*, and several others [4, 5].

Non-surgical removal of the microbial deposits, the subgingival debridement (instrumentation) is the standard in any cause-related periodontal therapy [6]. But a sole mechanical debridement may be insufficient in completely removing the causative biofilm [7] and may benefit from the use of an adjunctive antiseptic [8, 9]. The standard antiseptic in periodontal treatment is chlorhexidine digluconate (CHX) in a concentration of 0.12–0.2%, as a mouth wash for the home plaque control following periodontal treatment, but also to rinse the periodontal pocket right after mechanical debridement [10]. CHX shows good antibacterial properties and a long substantivity, although tooth surface discoloration is observed as side effect [11]. Further, it is cytotoxic to oral cells [12, 13], in vitro it may induce resistance in oral bacteria [13, 14], and it was found that CHX affects homoeostasis of oral microbiota and promotes selection of bacteria being resistant to common antibiotics in vivo [15].

An interesting alternative in periodontal therapy might be sodium hypochlorite (NaOCl)–based agents. They are a well-known irrigant in endodontic therapy [16]. In supportive periodontal therapy, NaOCl solution was tested and found as effective in reducing inflammation already in the early 1980s of the last century [17]. After a time of neglecting its positive properties in periodontal therapy, it was rediscovered about 10 years ago [18] when an amino acid (AA)–buffered slightly viscous NaOCl solution (AA-NaOCl gel) was introduced to the market. This formulation consists of two components, a diluted NaOCl solution and a viscous amino acid solution containing carboxymethyl cellulose as viscosity builder. The two components are mixed right before use in order to achieve a slightly viscous alkaline NaOCl solution for application in periodontal pockets. In vitro studies underlined the potential of the NaOCl gel. A dentine surface treated in vitro with AA-NaOCl gel and rinsed thereafter with sodium chloride solution did not affect cell viability of periodontal ligament fibroblasts, whereas cell adhesion and spreading were promoted [19]. In another in vitro study, we have found that AA-NaOCl gel was able to disaggregate biofilms [20] and that its principal mode of anti-biofilm action is not based on antibacterial activity.

Recently, clinical studies underlined a beneficial effect of adjunctive application of an AA-NaOCl gel in periodontal therapy [21, 22]. Combined with minimal invasive non-surgical periodontal therapy, the adjunctive use of the AA-NaOCl gel improved the clinical outcome with respect to the periodontal probing depth (PPD), clinical attachment loss, sites with PPD ≥ 5 mm, and bleeding on probing (BOP) positive sites [21]. In supportive periodontal therapy, the adjunctive use of AA-NaOCl gel was in favor to an application of a chlorhexidine or of a placebo gel regarding reduction of sites with BOP and pocket closure [22]. However, in peri-implant mucositis, the AA-NaOCl gel did not show significant additional benefit [23]. Here, it has to be noted that in both groups, a chlorhexidine gel was applied after instrumentation and no complete resolution of inflammation was reached [23].

The following in vitro study was aimed to answer the questions (i) how the activity of AA-NaOCl gel changes over time after mixing of the two components, (ii) how the concentration of NaOCl in the gel mixture affects its activity, (iii) if a lower pH of the gel mixture decreases its activity, and (iv) if the antibacterial and biofilm disaggregating activity remains when the second component (amino acids) is replaced by hyaluronic acid.

## Materials and methods

### Test material

The AA-NaOCl gel (PERISOLV (batch number 5149536); Regedent AG, Zurich Switzerland) was mainly used by mixing the two components (component 1, 0.95% NaOCl; component 2, amino acids plus additives) according to the manufacturer’s instructions, meaning by the “two-connected-syringe” system. Notwithstanding, in series (ii), the gel component of regular product was mixed with an equal volume of different freshly prepared NaOCl concentrations (0.95%, 0.8%, 0.6%, 0.4% NaOCl) by repeated pipetting of the mixture in Eppendorf tubes. In series (iv), besides the AA-NaOCl gel, NaOCl (0.5%) and hyaluronic acid (HA, hyadent BG, Regedent AG, containing 16 mg/ml cross-linked hyaluronic acid and 2 mg/ml natural hyaluronic acid) were used. As negative control served 0.9% w/v NaCl solution.

### Microorganisms

The following microorganisms were included:

1. *Porphyromonas gingivalis* ATCC 33277
2. *Tannerella forsythia* ATCC 43037
3. *Fusobacterium nucleatum* ATCC 25586
4. *Streptococcus gordonii* ATCC 10558
5. *Actinomyces naeslundii* ATCC 12104
6. *Parvimonas micra* ATCC 33270

The strains were precultivated on tryptic soy agar plates (Oxoid Ltd, Basingstoke, GB) with 5% of sheep blood. They were suspended in 0.9% w/v NaCl according to McFarland 4. Then, for all biofilm assays, a mixed suspension was prepared by adding one part of *S. gordonii* suspension, two parts of *A. naeslundii* suspension and each four parts of the other bacterial suspensions.

### Series (i): different times of mixing the components before application

The biofilm formation followed the protocols described before [20]. In short, three 96-well-plates were coated with a protein solution (1.5% bovine serum albumin) for 15 min. Biofilms using the multispecies mixture mixed with nutrient broth (Wilkins-Chalgren broth, Oxoid) in a ratio 1:9 consisting of six species were formed for 5 days. After 3.5 days, nutrient broth was exchanged and *P. gingivalis* and *T. forsythia* were added again.

At 5 days, the AA-NaOCl gel mixtures were prepared each 30 min, 20 min, 10 min, 5 min, and 2 min (considered immediate application) before application. The media on the biofilms were removed, and the biofilms were carefully washed with phosphate-buffered saline (PBS). Then, 25 µl of test substances was added to each of the four wells containing biofilm for the respective test substance. After 5 min, nutrient broth (225 µl) was added to dilute the test substance tenfold (mimicking the gingival crevicular flow), and the biofilms were analyzed after an additional 10 min of incubation.

From the 96-well plates, the supernatants were removed, and the remaining biofilms were carefully washed with 0.9% w/v NaCl. Each 200 µl of 0.9% w/v NaCl solution were added per well of the first plate. Then, the biofilms were removed from the surface by scraping supported by ultrasonication. The resulting biofilm suspension was mixed by pipetting, a serial dilution was made for each well, and the total colony-forming unit (cfu) counts were assessed. From the second plate, quantification of the biofilms was made after staining with crystal violet according to the recently published protocols [24], and from the third plate, the metabolic activity of the remaining biofilm on the surface was assessed using Alamar blue as a redox indicator according established protocols [25].

Additionally and independent of the biofilm experiments, the pH of the gel mixtures was determined immediately (2 min), 5 min, 10 min, 20 min, and 20 min after mixing.

### Series (ii): different NaOCl concentrations in the mixtures

The procedures were similarly as described for the first series. However, here the NaOCl concentration in the gel preparations differed. The first component contained 0.95%, 0.8%, 0.6%, and 0.4% NaOCl resulting in a concentration of 0.475%, 0.4%, 0.3%, and 0.2% NaOCl in the mixed gel. The mixing occurred immediately before its application on the biofilm, i.e., the NaOCl formulation with different NaOCl concentrations were applied on the biofilm within 2 min after mixing.

Again, the pH of the mixtures with the different NaOCl concentrations was determined immediately after mixing independent of the biofilm experiments.

### Series (iii): different pH of the mixtures

For this series, the mixing of the gels occurred again immediately before application. The pH of the gel mixture (with 0.95% NaOCl in the first component) was set right after mixing to pH 5, pH 7, and pH 9 by adding 0.1 M or 1 M HCl (without significantly altering the overall volume (less than 5%) and consequently NaOCl concentration). All other procedures were as described above.

### Series (iv): NaOCl gel mixture with hyaluronic acid

The test substances were AA-NaOCl gel, 0.5% NaOCl, 9 mg/ml HA (the commercial gel was mixed 1:1 with 0.9% w/v NaCl), and 0.5%NaOCl/9 mg/ml HA (the commercial gel was mixed 1:1 with 1% NaOCl). All test substances were adjusted to pH 12 before using. 

First micro-broth dilution technique was used for determination of MICs. A defined inoculum of the test strain was added to broth containing defined concentrations of test substances (starting from 10% of the final concentration in the respective formulation). After an incubation time of 42 h (18 h aerobes), the growth of microbes was analyzed by visual checking of turbidity. MIC represented the lowest concentration without visible turbidity.

Then, biofilms were formed, treated, and analyzed as described in series (i). However, all test substances were immediately applied after preparation (within 2 min). In addition to the analysis in series (i), DNA was visualized in biofilms. For that, biofilms were cultured on glass slides in 24-well plates using 150 µl of test substance and 1350 µl of nutrient media. Then, the DNA of the biofilm matrix was stained with 0.1% acridine orange solution (Merck KGaA, Darmstadt, Germany) as a general nucleic acid stain. Samples were examined by using fluorescent microscope with an objective lens having a 20-fold magnification (Olympus BX51, Tokyo, Japan).

### Statistical analysis

The MIC determinations were made in independent replicates. All biofilm experiments were made in two independent experiments in each with independent quadruplicates (eight independent results). Cfu counts (log10), biofilm quantity, and metabolic activity were compared with the help of Kruskal–Wallis test followed by Mann–Whitney test with Bonferroni correction using SPSS 28.0 (IBM Corporation, New York, NY, USA). *P* < 0.05 was considered as statistically significant.

## Results

### Series (i): different times of mixing the components before application

**Fig. 1:**
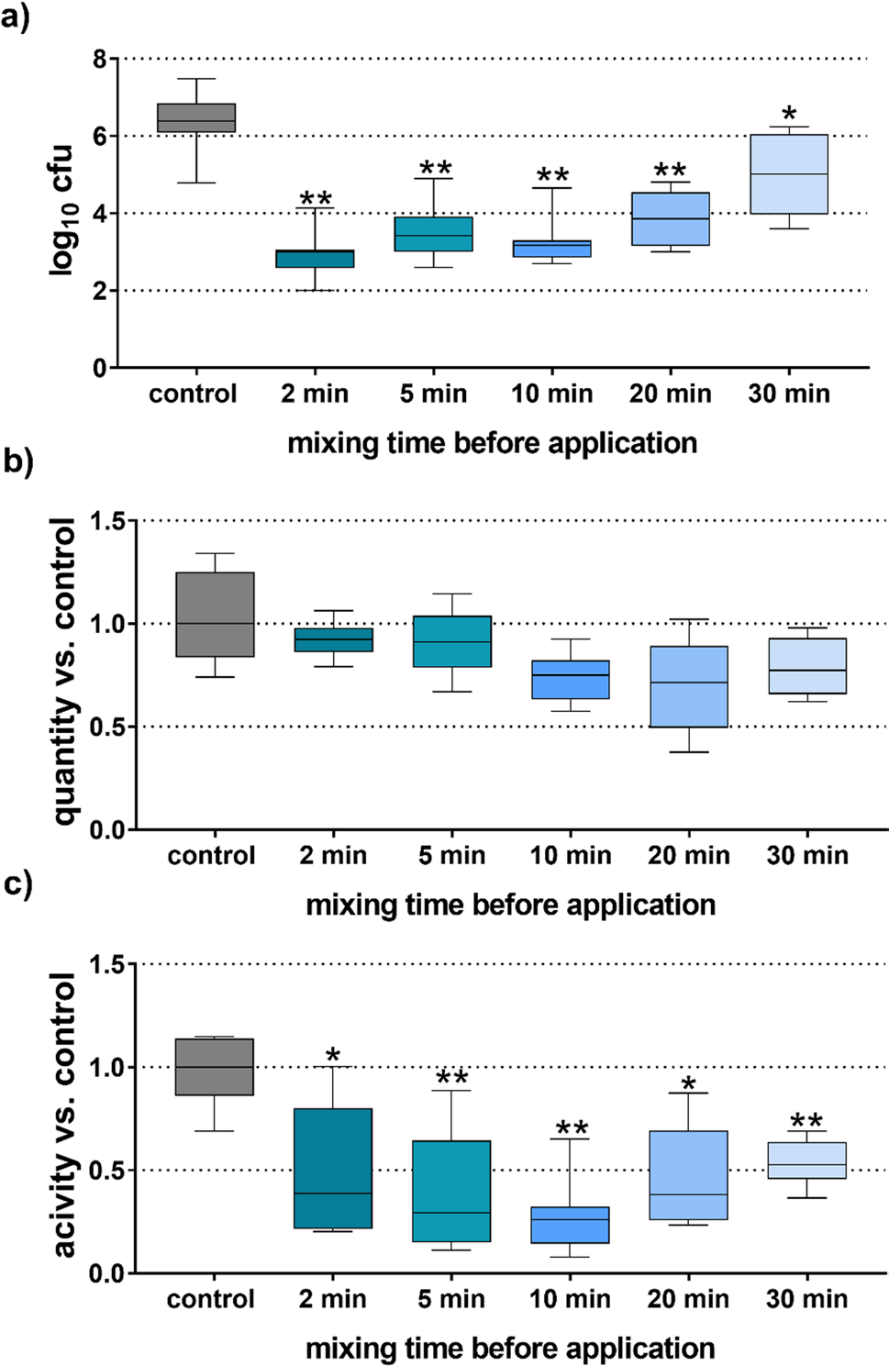
Cfu counts (**a**), biofilm quantity (**b**), and metabolic activity (**c**) of the residual biofilm related to the mixing time of the AA-NaOCl gel before application to the biofilm. */**p < 0.05/0.01 vs. control. (Presented are median, 25th and 75th percentiles, and range.)

The results confirmed an activity of the AA-NaOCl gel up to 30 min after mixing (except for 30 min (*p* = 0.025) all other times vs. control *p* < 0.001; Fig. 1a). However, when waiting 30 min after mixing before application, the difference of the median cfu reduction compared to the negative control was only 1.37 log10. At all other mixing time points, the difference was at least 2.5 log10 cfu. Application of the mixture up to 10 min after mixing decreased the cfu counts significantly more than waiting 30 min (vs. 2 min *p* < 0.001, 10 min *p* = 0.010). The measured mass of the remaining biofilm tended to be less than for the control when the formulation was applied 10–20 min after mixing but not with no statistical significances (Fig. 1b). The metabolic activity as an indicator for the vitality of the remaining biofilm was significantly lower for all mixing time points compared to the control (2 min *p* = 0.015; 20 min *p* = 0.010, other times vs. control *p* < 0.001) with no significant differences between the different mixing time points (Fig. 1c).

In addition, the pH of the NaOCl formulation used for the experiments was measured at the time of the application. Results show that shortly after mixing, the pH slightly increased to pH 12.5, and after 5 min, it started to slightly decrease. Twenty minutes after mixing, the pH decreased to pH 12.0 and after 30 min to pH 11.5.

### Series (ii): different NaOCl concentrations in the mixtures

**Fig. 2:**
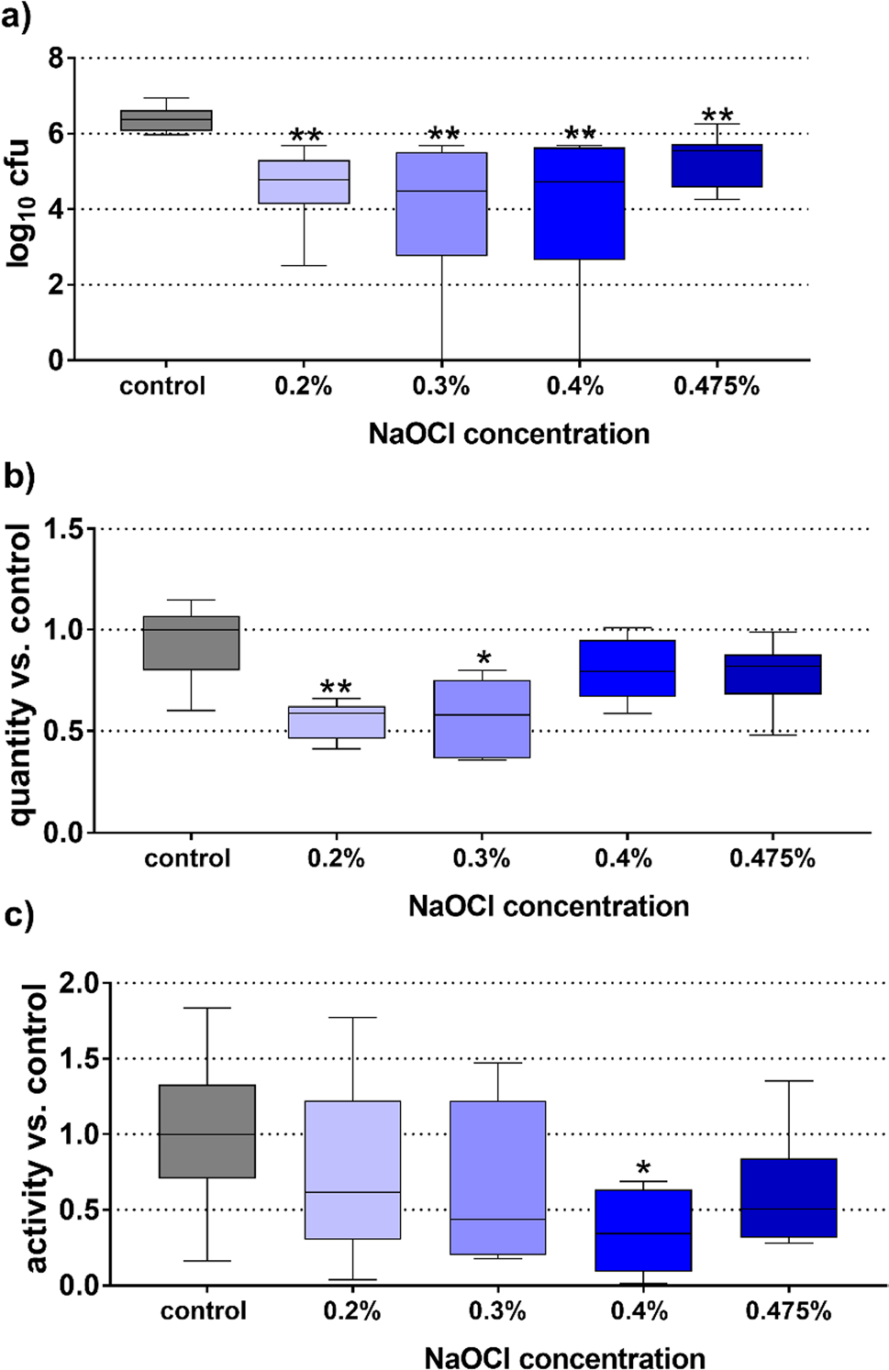
Cfu counts (**a**), biofilm quantity (**b**), and metabolic activity (**c**) of the residual biofilm related to the NaOCl concentration of the formulation. */**p < 0.05/0.01 vs. control. (Presented are median, 25th and 75th percentiles, and range.)

Regarding the cfu counts (Fig. 2a), all mixtures significantly reduced the cfu counts (each *p* < 0.001 vs control). The mixture containing 0.3% NaOCl was most active (− 1.89 log10 cfu). The reductions for the 0.2% and 0.4% NaOCl formulation were 1.60 log10 and 1.65 log10 cfu. The values for the biofilm quantity (Fig. 2b) were found to be significantly lower for the formulations containing 0.2% and 0.3% NaOCl than for the control (*p* = 0.004, *p* = 0.012). In terms of reduction of metabolic activity, only the 0.4% NaOCl containing formulation reduced the biofilm metabolic activity (*p* = 0.040; Fig. 2c).

Also in this series, additionally and independent of the biofilm experiments, the pH of the mixtures with the different NaOCl concentrations was determined immediately after mixing. Results show that the pH only very slightly decreases with decreasing NaOCl concentration and remains in the range of pH 12 – 12.25.

### Series (iii): different pH of the mixtures

**Fig. 3:**
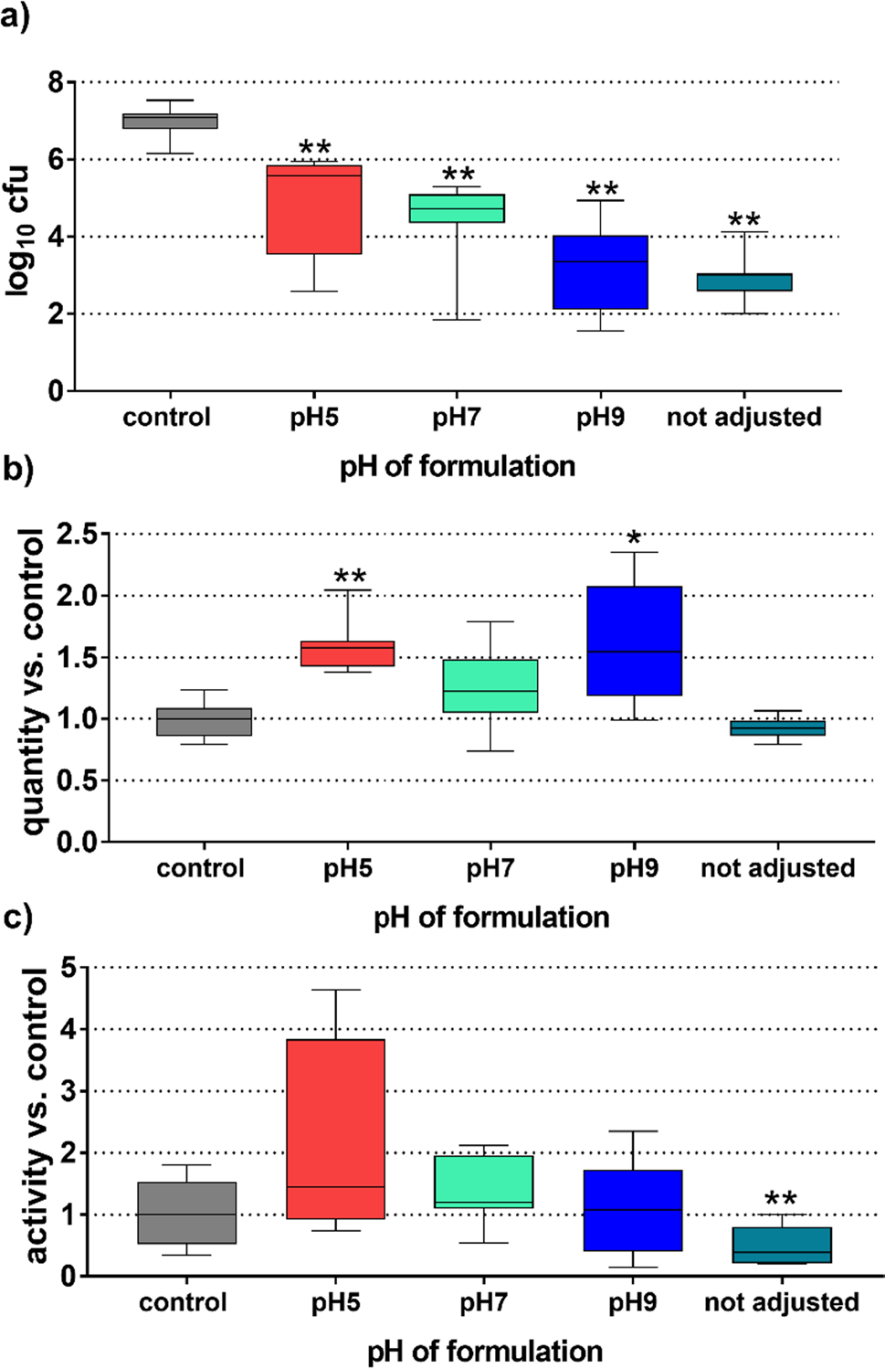
Cfu counts (**a**), biofilm quantity (**b**), and metabolic activity (**c**) of the residual biofilm related to the pH of the AA-NaOCl gel. */**p < 0.05/0.01 vs. control. (Presented are median, 25th and 75th percentiles, and range.)

The cfu counts in the residual biofilm decreased statistically significantly after adding the NaOCl gels set to the different pH (all *p* < 0.001; Fig. 3a). But their numbers decreased more at pH 9 and at a not adjusted pH compared to pH 5 (*p* = 0.028; *p* < 0.001). Regarding the biofilm quantity (Fig. 3b), an increased quantity vs. control was found at pH 5 (*p* < 0.001) and pH 9 (*p* = 0.012). For the adjusted NaOCl gels, there was no difference vs. control regarding the biofilm activity (Fig. 3c).

### Series (iv): NaOCl gel mixture with hyaluronic acid

The MICs against planktonic single strains were measured first. HA did not act growth inhibitory on the tested strains. But in mixtures with NaOCl, HA did not negatively influence the MIC values of NaOCl. For four of the six tested bacteria the results of the MIC values were in accordance between NaOCl and AA-NaOCl (5% AA-NaOCl gel contains about 0.025% NaOCl; Table 1)*.*

**Table 1:** MIC values of sodium hypochlorite, hyaluronic acid, and respective formulations against selected oral microorganisms

	NaOCl (%)	Hyaluronic acid (mg/ml)	Hyaluronic acid (mg/ml)/NaOCl (%)	AA-NaOCl gel (%)
*Porphyromonas gingivalis* ATCC 33277	0.025	> 0.7	0.35/0.025	5
*Tannerella forsythia* ATCC 43037	0.025	> 0.7	0.35/0.025	5
*Fusobacterium nucleatum* ATCC 25586	0.025	> 0.7	0.35/0.025	5
*Streptococcus gordonii* ATCC 10558	0.025	> 0.7	0.175/0.0125	> 10
*Actinomyces naeslundii* ATCC 12104	0.0125	> 0.7	0.175/0.0125	0.625
*Parvimonas micra* ATCC 33270	0.025	> 0.7	0.175/0.0125	5

**Fig. 4:**
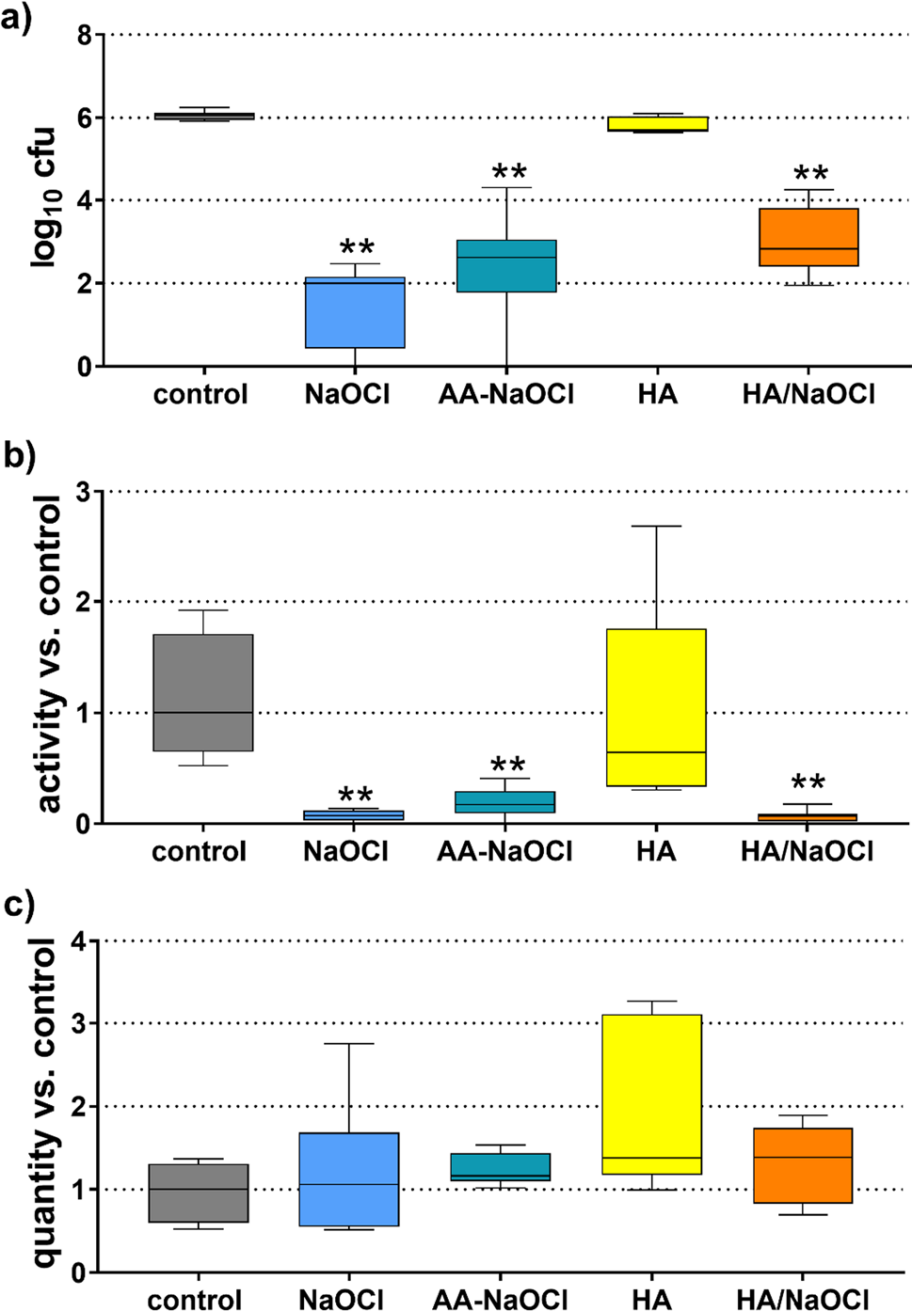
Activity of AA-NaOCl gel, hyaluronic acid (HA), sodium hypochlorite (NaOCl), hyaluronic acid/sodium hypochlorite mixture (HA/NaOCl) on colony-forming unit (cfu; **a**), quantity (**b**), and metabolic activity (**c**) of a 5-day-old multi-species biofilm. **p < 0.01 vs. control. (Presented are median, 25th and 75th percentiles, and range.)

The cfu counts in biofilms decreased most after applying NaOCl (− 4.06 log10 cfu; *p* < 0.001). The HA/NaOCl formulation and the AA-NaOCl gel reduced bacterial counts by 3.23 log10 cfu and 3.43 log10 cfu vs. control (both *p* < 0.001; Fig. 4a). All compounds did not change statistically significantly the biofilm quantity; adding HA increased the biofilm quantity by trend, (Fig. 4b). The metabolic activity of the biofilms was reduced by all NaOCl-containing compounds (each *p* < 0.001; Fig. 4c). In all three analyses, there was no statistical significant difference between HA/NaOCl formulation and the AA-NaOCl gel.

In addition, the pH of the used substances was measured, those of HA was around pH 6.4, of NaOCl pH 11.5, and of the HA/NaOCl mixture pH 9.0.

## DNA staining

**Fig. 5:**
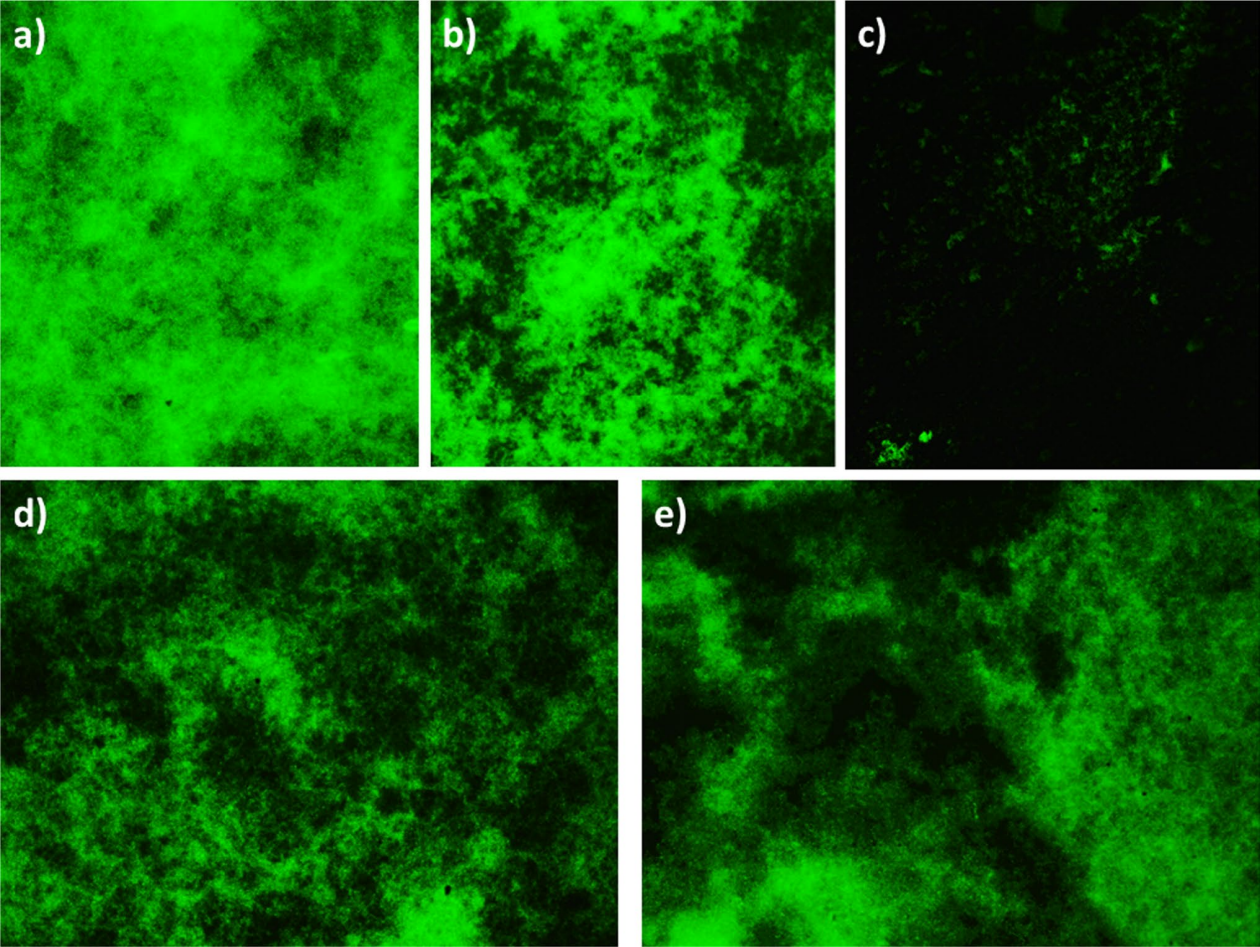
DNA staining of a 5-day-old biofilm without (**a**) and with addition of hyaluronic acid (**b**), sodium hypochlorite (**c**), AA-NaOCl gel (**d**), and hyaluronic acid/sodium hypochlorite mixture (**e**)

The densest stained biofilm was visible in the controls (Fig. 5a). The biofilm after applying HA (Fig. 5b) was less densely stained than the control. Sodium hypochlorite eliminated bacteria and seemed to degrade matrix of the biofilm (Fig. 5c). After adding AA-NaOCl gel (Fig. 5d) or HA/NaOCl (Fig. 5e), the biofilms were less stained than the control but more stained compared to NaOCl alone. No clear differences were observed between the AA-NaOCl gel and the HA/NaOCl mixture.

## Discussion

The purpose of the present in vitro study was to get deeper knowledge on the influence of different parameters on the anti-biofilm activity of an AA-NaOCl gel designated as an adjunctive in periodontal treatment. The studies’ parameters were the time after mixing before potential application, the pH, and the chemical composition of the formulation.

Periodontal disease is the biofilm-induced and host-mediated inflammation and destruction of the tooth surrounding tissue [26]. The crucial part of the therapy is the subgingival instrumentation, i.e., the mechanical removal of the supragingival biofilm and the mineralized deposits, to enable resolution of the inflammatory reaction and reestablishment of the adjunct tissues [9]. In the guideline of the European Federation of Periodontology however, the application of adjunctive antiseptics is not clearly recommended nor not recommend, but relied only on chlorhexidine. Nevertheless, CHX is not capable in degrading the extracellular matrix of the biofilm [27, 28]. Therefore, remnants still cover the surface and avoid reattachment of the tissue. In our study NaOCl 0.3% showed both a significant reduction of the total biofilm mass and a decrease in viability of the bacteria.

Before application, the AA-NaOCl gel has to be prepared by mixing. This raises the question for the best time-point which would also suit to the treatment protocol at all. To enable some flexibility in the application, the stability of the mixture is a precondition. The second component of the AA-NaOCl gel contains the amino acids glutamic acid, leucine, and lysine. In a recent study, NaOCl was mixed with different amino acids which generated N-chloro-amino acids [29]. Mixing glutamic acid or leucine with NaOCl produced two peaks, whereas the mixture with lysine resulted in a typical monochloramine peak [29]. The product of lysine with NaOCl was investigated further; it was moderately stable [29]. Our mixing time point experiments showed that the AA-NaOCl gel was capable of statistically significantly reducing cfu counts (numbers of the viable bacteria) up to 30 min after mixing. But only up to 20 min after mixing a log10 reduction above 2 was detectable. Equally, the NaOCl formulation reduced significantly biofilm metabolic activity up to 30 min after mixing. Based on these experimental data, it can be concluded that the formulation can be applied up to 30 min after mixing, preferentially within 20 min after mixing.

Sodium hypochlorite has the potential to cause toxic reactions due to its oxidizing capacity and its high pH [30]. Viabiltiy of human gingival fibroblasts decreased to about 35%, when 0.1% NaOCl solution was applied for 5 min [31]. But this should be also related to other antiseptics commonly used in dental therapy. Low concentrated NaOCl (0.05%) did not affect dental mesenchymal stem cell survival after 10 min, whereas low concentrated CHX (0.02%) caused a strong cytotoxicity [32]. Further, computational prediction did not find any mutagenic, tumorigenic irritant and reproductive toxicity [33].

Anyway it raised the question if the anti-biofilm activity can be kept when the concentration of NaOCl is reduced. Our results suggest that using a lower NaOCl concentration does not necessarily result in a reduced activity on biofilms. Lower NaOCl concentrations may be equally or even more active against an existing biofilm than the regularly used concentration. However, it has to mentioned that in this series in contrast to other experiments, the formulation containing regular NaOCl concentration (0.45%) did not result in a significant cfu reduction compared to control. This might be explained by the fact that the mixing of the AA containing gel component with the NaOCl component of different concentrations was done by pipetting and not by the “two-connected-syringe” system and following less effective. A concentration-dependent effect of NaOCl solutions was reported in dialysis catheters with lower concentrated NaOCl (0.005–0.1%) and a treatment time of 30 min [34].

The followed pH experiments indicated that a reduced pH results in a reduced activity of the NaOCl formulation on the biofilm and that a higher pH (pH 9 or above) is of importance for the activity of the NaOCl formulation. Our results contrast another study where NaOCl at pH 5 was more active than at pH 12 [35]. There, an in situ biofilm was created in root canals; bacterial cell viability was determined by live-dead staining [35]. A study on dialysis equipment did not find a clear influence of pH (8.5–11) on anti-biofilm activity [34].

As mentioned before, the AA-NaOCl gel is a mixture of two components. The AA-component provides viscosity for the formulation. It might generate monochloramines to a certain extent. Monochloramines can act as immunomodulators. They inhibited TNFα secretion in a murine macrophage cell line but reduced also dependent on the concentration of chloramines the viability of the cells [29]. In our recent in vitro study, the AA-component had direct antibacterial activity against some Gram-negative bacteria; it slightly inhibited biofilm formation but did not affect cfu counts of an existing biofilm [20]. Others tested the potential of D-amino acids (a mixture of three AA among them D-leucine) to inhibit biofilm formation of *Enterococcus faecalis* and found less biofilm formation, to a limited degree also in the presence of low concentrated NaOCl [36]. Nevertheless, there is the question if the AA component could be replaced by another one. Comparing the NaOCl component and the NaOCl gel formulation showed at least the same activity of the NaOCl alone as the gel formulation against biofilms [20]. In the present study, the second component was replaced by a cross-linked hyaluronic acid formulation. It functioned as a viscosity builder which may indicate that the HA was not degraded. Degraded hyaluronic acid loses its dynamic viscosity [37]. Hyaluronic acid is a molecule of interest for many treatment options in medicine as ophthalmology, rheumatology and dermatology [38]. Having a high molecular weight, it has anti-inflammatory and immunosuppressive properties; degraded and having lower molecular weight, it may stimulate an inflammatory response [39]. In periodontitis treatment, adjunctive hyaluronic acid was applied in non-surgical and surgical therapies; recently, a systematic review showed an additional benefit in the clinical outcome [40]. NaOCl combined with the HA formulation was at least as active as the AA-NaOCl-gel formulation in biofilm disaggregation. It is of interest to further study the combination, e.g., in different concentrations of the NaOCl and HA component each and also in different modes of application. Clinically a retrospective case series analysis underlined the potential of the combination when HA was used sequentially after the NaOCl gel application [41].

The present in vitro study focused on the anti-biofilm activity of a NaOCl gel formulation. Of clinical relevance is the finding that the AA-NaOCl gel formulation can be mixed up to 20 min prior to application. Further, the study indicates that the composition of the NaOCl gel formulation can be optimized. This study did not consider aspects of interaction of bacteria with host cells, adhesion of periodontal cells to dentin surfaces, cytotoxicity, or immunomodulation. This might be a limitation and should be investigated further together with a potential modification of the NaOCl gel.

## Data Availability

The data presented in this study are available on request from the corresponding author.
